# Bundesweiter Vergleich zu heilkundlichen Maßnahmen durch Notfallsanitäterinnen und Notfallsanitäter

**DOI:** 10.1007/s00101-025-01509-6

**Published:** 2025-02-24

**Authors:** Michael S. Dittmar, Marina Kraus, Bernhard M. Graf

**Affiliations:** 1 Klinik für Anästhesiologie, Universitätsklinikum Regensburg, Franz-Josef-Strauss-Allee 11, 93053 Regensburg, Deutschland; 2 Klinik für Innere Medizin II, Klinikum St. Marien Amberg, Amberg, Deutschland

**Keywords:** Rettungsdienst, Delegation, SOP, Opioidanalgetika, Regionale Unterschiede, Emergency medical services, Delegation, Standard operating procedures, Analgesics, opioid, Regional differences

## Abstract

**Hintergrund:**

Die heilkundliche Tätigkeit durch Notfallsanitäterinnen und Notfallsanitäter ohne notärztliche Anwesenheit basiert v. a. 1.) auf einer Delegation durch z. B. die Ärztliche Leitung Rettungsdienst oder 2.) auf eigenverantwortlicher Heilkundeausübung nach § 2a Notfallsanitätergesetz. Beide Möglichkeiten unterscheiden sich u. a. hinsichtlich der Verantwortung für die Indikationsstellung. Diese Arbeit gibt erstmals einen bundesweiten Überblick, wer welche Verantwortung im Rahmen der Behandlungsvorgaben für Notfallsanitäterinnen und Notfallsanitäter trägt.

**Material und Methoden:**

Die Behandlungsalgorithmen zu 5 Krankheitsbildern wurden für alle Bundesländer hinsichtlich ihrer geografischen Gültigkeit, der Deklaration sowie des objektiven Charakters als Delegation der Ärztlichen Leitung Rettungsdienst oder entsprechend verantwortlicher Ärztinnen oder Ärzte (ÄLRD-Delegation) oder Heilkundeausübung (§ 2a NotSanG) und der Erstreckung auf Betäubungsmittel ausgewertet. Die Datenerhebung fand im Zeitraum von Dezember 2020 bis Juni 2022 statt.

**Ergebnisse:**

Es wurden 112 Algorithmen mit 403 Einzelmaßnahmen analysiert. Für 11 Bundesländer wurden landesweit gültige, in 5 Ländern regional abweichende Behandlungsvorgaben gefunden. Der ÄLRD-Delegations- oder § 2a NotSanG-Status war in lediglich 40 % der einzelnen Maßnahmen explizit deklariert. Diese Deklaration zeigte in 93 % eine Übereinstimmung mit dem objektiven Charakter der Maßnahme. Eine eigenständige oder eigenverantwortliche Betäubungsmittelgabe durch NotSan ist in 6 Ländern vorgesehen.

**Diskussion:**

In der Mehrheit der für NotSan vorgesehenen Maßnahmen ist nicht ersichtlich, ob diese in ÄLRD-Delegation oder nach § 2a NotSanG angewendet werden sollen. Eine entsprechende Deklaration durch die Ersteller könnte hier für mehr Klarheit bezüglich der Verantwortlichkeiten sorgen. Sowohl eine ÄLRD-Delegation als auch eine Betäubungsmittelgabe ohne Arztanwesenheit ist nicht in allen Bundesländern etabliert. Aufgrund der sich stetig weiterentwickelnden Rechtslage stellen sich die untersuchten Endpunkte in einzelnen Regionen mittlerweile anders dar.

**Graphic abstract:**

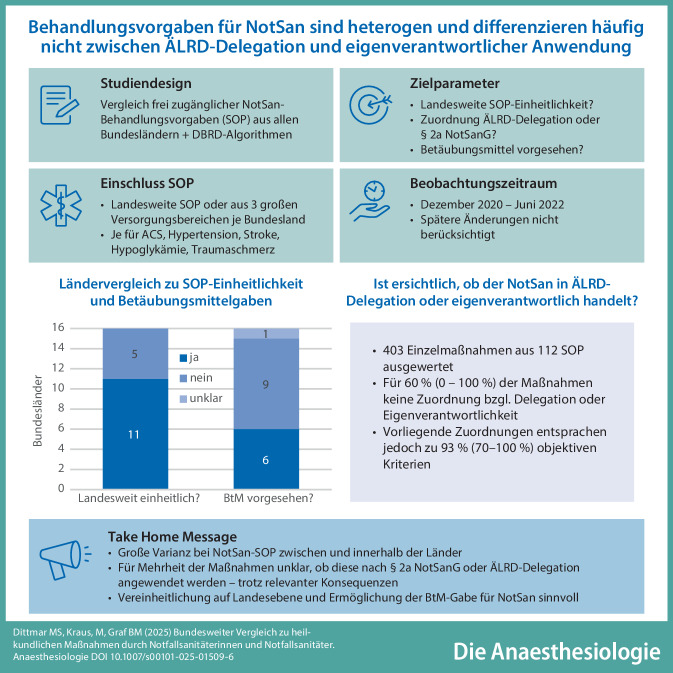

## Abstract (en)

**Background:**

The medical treatment by emergency paramedics without the presence of an emergency physician in Germany is based primarily on 1) delegation by the Medical Director of Emergency Medical Services (AELRD) or 2) the independent practice of medicine according to § 2a of the German Federal Emergency Paramedic Act (NotSanG). Both possibilities differ with respect to the responsibility for the diagnosis and treatment decision. This article provides for the first time a nationwide overview of who bears which responsibilities for treatment specifications for emergency paramedics.

**Material and methods:**

The treatment algorithms for five clinical pictures were evaluated for all German federal states with respect to their geographical validity, the declaration and objective character as the delegation by the AELRD or the practice of medicine (§ 2a NotSanG) and the extension to narcotics. The data collection took place between December 2020 and June 2022.

**Results:**

In this study 112 algorithms with 403 individual measures were analyzed. For 11 German states unified treatment specifications were found and in 5 federal states treatment specifications differing from region to region were identified. The AELRD delegation or § 2a NotSanG status was explicitly declared in only 40% of the individual measures. This declaration was consistent with the objective character of the measure in 93%. An independent narcotics administration by paramedics is established in six states.

**Conclusion:**

In the majority of measures intended for paramedics it is not obvious whether they are to be applied according to AELRD delegation or § 2a NotSanG. Such a declaration by the preparers could provide more clarity with respect to the responsibilities. Both an AELRD delegation and the administration of narcotics by paramedics without the presence of a physician are not established in all German states. Due to the continuously developing legal situation, the investigated endpoints may have changed in the meantime in some regions.

## Infobox Nicht an NotSan delegierbare heilkundliche Maßnahmen. (Nach Dittmar et al. [1])

- Endotracheale Intubation und invasive Beatmung
- Nichtinvasive Beatmung
- Thoraxentlastungspunktion
- Koniotomie
- Defibrillation/Kardioversion ohne Sedierung
- Kardioversion mit Sedierung
- Anwendung eines externen Schrittmachers
- Diagnosestellung
- Geburtsbegleitung
- Neugeborenenversorgung

## Hintergrund und Fragestellung

Notfallsanitäterinnen und Notfallsanitäter (NotSan) übernehmen im bundesdeutschen Rettungsdienst in unterschiedlichen Konstellationen heilkundliche Maßnahmen, ohne dass sich eine Notärztin oder ein Notarzt (NA) am Einsatz befindet. Insbesondere ist dabei die eigenständige Übernahme von Maßnahmen in Delegation durch die Ärztliche Leitung Rettungsdienst (ÄLRD) oder entsprechend verantwortliche Ärztinnen oder Ärzte (im Folgenden verkürzt als ÄLRD-Delegation bezeichnet) abzugrenzen von der eigenverantwortlichen Ausübung der Heilkunde nach § 2a des Notfallsanitätergesetzes (NotSanG) in Situationen, in denen bereits vor dem Arztkontakt ein sofortiges Eingreifen erforderlich ist, um Lebensgefahr oder bleibende Folgeschäden abzuwenden.

Bei der erstgenannten ÄLRD-Delegation definiert die ÄLRD die Indikation sowie Art und Umfang der delegierten Maßnahmen und übernimmt somit die Anordnungsverantwortung. Die NotSan sind für die sachgerechte Umsetzung der ÄLRD-Delegation verantwortlich. Dabei soll nach Ansicht des Wissenschaftlichen Dienstes des Bundestags die Delegation so gestaltet sein, dass NotSan keine wesentlichen eigenen Entscheidungsspielräume ausüben [2]. Nach der Gesetzeslage ist es nicht erforderlich, dass eine akute Lebensbedrohung oder die Gefahr wesentlicher Folgeschäden vorliegt, was eine breitere Anwendbarkeit der Delegation auch jenseits hochkritischer Notfälle ermöglicht. Abhängig vom Delegationsinhalt ist es nicht zwingend notwendig, den Patienten bzw. die Patientin ärztlich vorzustellen. Bei Vorliegen der Voraussetzungen ist der Algorithmus in vollem Umfang durch NotSan umzusetzen. Medikamentendosierungen und andere Vorgaben sind verbindlich. Im NotSanG ist diese Konstellation als Ausbildungsziel in § 4 Abs. 2 Nr. 2c abgebildet.

Daneben ist der NotSan unter bestimmten Bedingungen zur eigenverantwortlichen Ausübung der Heilkunde berechtigt. Paragraf 2a NotSanG führt hierzu aus:

„Bis zum Eintreffen der Notärztin oder des Notarztes oder bis zum Beginn einer weiteren ärztlichen, auch teleärztlichen, Versorgung dürfen Notfallsanitäterinnen und Notfallsanitäter heilkundliche Maßnahmen, einschließlich heilkundlicher Maßnahmen invasiver Art, dann eigenverantwortlich durchführen, wenn

1. sie diese Maßnahmen in ihrer Ausbildung erlernt haben und beherrschen und

2. die Maßnahmen jeweils erforderlich sind, um Lebensgefahr oder wesentliche Folgeschäden von der Patientin oder dem Patienten abzuwenden.“

Auch in diesen Fällen können die NotSan auf Behandlungsalgorithmen, Checklisten oder Ähnliches zurückgreifen, entscheiden jedoch vollständig selbst über Art und Umfang der Therapie. Dementsprechend sind bestehende Vorgaben oder Therapieempfehlungen nicht verbindlich. Die NotSan tragen die gesamte Verantwortung für ihr Handeln und haben stets sorgfältig abzuwägen, ob eine infrage kommende Maßnahme unter Würdigung aller Umstände im Einzelfall (z. B. erwartetes Eintreffen des NA, erwarteter Nutzen und Risiken der Intervention, eigene Erfahrung mit der fraglichen Maßnahme, widerstreitende Therapieziele bei Erkrankungs‑/Verletzungskombinationen, Patientenwünsche etc.) tatsächlich entscheidend zur Lebensrettung bzw. zur Abwendung von Folgeschäden beitragen wird. Die schnellstmögliche Zuführung zu einer ärztlichen Behandlung ist sowohl laut Gesetz als auch aufgrund des definitionsgemäßen Patientenzustands in jedem Fall erforderlich. Schon aufgrund der Eingangsvoraussetzungen ist in aller Regel die frühzeitige Nachforderung eines NA angezeigt. Die Berufsausbildung für diese verantwortungsvolle Tätigkeit ist in § 4 Abs. 2 Nr. 1c NotSanG geregelt. Demnach sind bestimmte für die eigenverantwortliche Patientenversorgung in lebensbedrohlichen Notfällen erforderliche Kompetenzen bis zum „Beherrschen“ im Rahmen der Ausbildung zu vermitteln.

Nicht weiter eingegangen werden soll auf Aufgaben, welche NotSan im Rahmen einer Einzelfalldelegation durch einen anwesenden NA oder Tele-NA übernehmen.

Obwohl sich Voraussetzungen und Verantwortlichkeiten der ÄLRD-Delegation wesentlich von der eigenverantwortlichen Heilkundeausübung nach § 2a NotSanG unterscheiden, bleibt in der Praxis häufig unklar, welche der beiden Rechtfertigungen bei der Erstellung von Behandlungsvorgaben für NotSan oder bei der praktischen Tätigkeit der NotSan im Einzelfall herangezogen werden. Bis dato existiert kein Überblick, wie Behandlungsvorgaben und -empfehlungen für NotSan in der Bundesrepublik in Bezug auf die Verteilung der Verantwortlichkeiten einzuordnen sind, und ob sich diese auch auf die Verabreichung von Betäubungsmitteln erstrecken.

Diese Arbeit stellt eine systematische, bundesweite Analyse dazu dar, inwieweit die in Behandlungsalgorithmen und -Checklisten für NotSan enthaltenen Maßnahmen in den einzelnen Bundesländern

- landesweit einheitlich vorgegeben sind,
- als von der ÄLRD delegiert oder als eigenverantwortlich anzuwenden deklariert sind,
- sich nach objektiven Maßstäben am ehesten einer ÄLRD-Delegation oder einer eigenverantwortlichen Tätigkeit zuordnen lassen,
- eine Übereinstimmung zwischen den beiden oben genannten Aspekten besteht,
- die Gabe von Betäubungsmitteln durch NotSan umfassen, sowie
- ob die ÄLRD-Delegation gesetzlich implementiert ist.

## Studiendesign und Untersuchungsmethoden

Über eine Google-Internetrecherche mit den Suchbegriffen „Algorithmen“, „Delegation“, „Handlungsempfehlungen“, „Maßnahmen Notfallsanitäter“, „Erweiterte Versorgungsmaßnahmen (EVM)“ „Standard operating procedure (SOP)“, „Rettungsdienst“, jeweils in Kombination mit den einzelnen Bundesländern, den Kreisverbänden des Deutschen Roten Kreuzes bzw. dem Stichwort „Feuerwehr“ wurden Behandlungsvorgaben für NotSan identifiziert. Zusätzlich wurde die verfügbare Literatur über eine Internetrecherche sowie anhand von Quellenangaben aus vorhandenen Publikationen ausgewertet. Sofern keine landesweit einheitlich geltenden Vorgaben existierten (oder neben landesweiten Ausarbeitungen noch mindestens 2 Rettungsdienstbereiche (RDB) mit abweichenden Festlegungen gefunden werden konnten), wurden pro Bundesland die für die 3 RDB bzw. Organisationseinheiten mit der höchsten Einwohnerzahl verfügbaren eingeschlossen, darunter – soweit vorhanden – die mit dem landesweiten Gültigkeitsanspruch. Zusätzlich wurden die Behandlungsalgorithmen des Deutschen Berufsverbands für den Rettungsdienst (DBRD) [3] analysiert.

Für jedes Bundesland bzw. eingeschlossenen Rettungsdienstbereich flossen die Algorithmen für die 5 Notfallsituationen akutes Koronarsyndrom, hypertensive(r) Notfall/Krise, Schlaganfall, Hypoglykämie und traumatisch bedingte Schmerzzustände in die Auswertung ein. Aus diesen Algorithmen wurden dann die einzelnen Maßnahmen bzw. Medikamentengaben extrahiert und nach den im Folgenden dargestellten Maßstäben betrachtet. Die Verabreichung mehrerer Medikamente zählte dabei pro Applikationsweg als eine singuläre Maßnahme, mit Ausnahme von Betäubungsmittelgaben, welche gesondert berücksichtigt wurden.

Die Datenerhebung fand im Zeitraum vom Dezember 2020 bis Juni 2022 statt. Ein Ethikvotum ist nach den Regularien der zuständigen Ethikkommission Regensburg nicht erforderlich [4]. Rohdaten stellen die Autoren auf Anforderung zur Verfügung.

### Gesetzgeberische Voraussetzungen für die ÄLRD-Delegation

Zur Beantwortung der Frage nach der gesetzlichen Verankerung der ÄLRD-Delegation erfolgte eine Durchsicht der Rettungsdienstgesetze und -verordnungen der 16 Bundesländer, wobei zwischen gesetzlich, untergesetzlich (Regelung in Rechtsverordnungen o. Ä.) und nicht vorhanden unterschieden wurde.

### Geografische Gültigkeit

Auf Basis der Internetrecherche wurde ausgewertet, ob landesweit einheitliche oder regional differierende Behandlungsvorgaben vorlagen. Falls trotz Vorhandensein landesweit übergreifender Vorgaben mindestens 2 RDB mit abweichenden Algorithmen gefunden wurden, werteten die Autoren dies als regional differierend.

### Verabreichung von Betäubungsmitteln durch NotSan

Gleichzeitig fragten wir, ob die Verabreichung von dem Betäubungsmittelgesetz unterliegenden Opioidanalgetika für NotSan vorgesehen ist, und wenn ja, in welcher deklarierten Konstellation (§ 2a NotSanG-Maßnahme, ÄLRD-Delegation, beides, unklar).

### Deklaration und objektivierbarer Charakter als § 2a-Maßnahme oder ÄLRD-Delegation

Es wurde für jede einzelne eingeschlossene Tätigkeit erhoben, ob diese die Ersteller der Behandlungsvorgaben explizit als eigenverantwortlich zu indizierende und anzuwendende Maßnahme/Medikamentengabe nach § 2a NotSanG („§ 2a-Maßnahme“) oder als durch die ÄLRD delegierte und eigenständig umzusetzende Handlung („ÄLRD-Delegation“) deklariert war.

Zusätzlich erfolgte für jede Einzelmaßnahme eine Einordnung des Charakters als § 2a-Maßnahme oder ÄLRD-Delegation nach objektivierbaren Gesichtspunkten. Hierzu kam jeweils eine selbst entwickelte Punktematrix zur Anwendung (Tab. 1). Aus den 5 Aspekten Deklaration durch den Ersteller, Verpflichtung zur Notarztbeteiligung, ÄLRD-Verantwortung, Voraussetzung einer Lebensbedrohung/Gefahr wesentlicher Folgeschäden, Ermessensspielraum für den NotSan und grundsätzliche Delegierbarkeit der Tätigkeit wurden Punkte für den Charakter einer § 2a-Maßnahme bzw. einer ÄLRD-Delegation gesammelt, welche miteinander verrechnet werden. Bei unklarer Ausprägung wurden 0 Punkte vergeben. Die Summe der Punkte konnte Werte von −5 bis +4 annehmen. Werte zwischen −5 und −2 wurden als Beleg für einen § 2a-Maßnahmen-Charakter gewertet, Punktwerte im Bereich von +2 bis +4 als Charakter einer ÄLRD-Delegation. Summenwerte von −1 bis +1 wurden als uneindeutig eingestuft.

**Tab. 1:** Punktesystem zur Bestimmung des Charakters als Maßnahme nach § 2a NotSanG („§ 2a-Maßnahme“) bzw. als Delegation der Ärztlichen Leitung Rettungsdienst („ÄLRD-Delegation“). Jede der 403 identifizierten Einzelmaßnahmen wurde nach dieser Matrix getrennt bewertet. Ein Punktwert von −5 bis −2 spricht für eine § 2a-Maßnahme, Werte von +2 bis +4 für eine ÄLRD-Delegation. Der Ergebnisbereich −1 bis +1 wird als uneindeutig gewertet

Aspekt	Argument für § 2a-Maßnahme (−1 Punkt)	Argument für ÄLRD-Delegation (+1 Punkt)	Uneindeutig
Einordnung als § 2a-Maßnahme/ÄLRD-Delegation vorgegeben?	Als § 2a-Maßnahme deklariert: −1	Als ÄLRD-Delegation deklariert: +1	0
Notarztbeteiligung?	Explizit mit verpflichtender Notarztbeteiligung: −1	Ohne Notarztbeteiligung möglich: +1	0
Vom ÄLRD vorgegeben und verantwortet?	Nein: −1	*n.* *a.*	0
Situation mit Lebensbedrohung/Gefahr scherwiegender Folgeschäden?	*n.* *a.*	Nein: +1	0
Ermessensspielraum für NotSan bei Therapieentscheidung (Befundgrenzen, Dosierung etc.)?	Ja: −1	Nein: +1	0
Tätigkeiten vollständig delegierbar?	Nein: −1	*n.* *a.*	0

*n.* *a*. kann nicht als Argument für die entsprechende Entität in der Spalte herangezogen werden

Während die Autoren die ersten 4 Aspekte gemeinschaftlich bewerteten, wurde für den Aspekt der Delegierbarkeit eine Umfrage unter den bayerischen ÄLRD herangezogen [1]. Es wurde ein Minuspunkt als Argument für den Charakter als § 2a-Maßnahme vergeben, sofern die zu bewertende Maßnahme als im Konsens nicht delegierbar eingestuft war (Infobox).

Zu jeder bewerteten Maßnahme erfolgte im nächsten Schritt ein Vergleich zwischen festgestelltem Charakter und der Deklaration durch die Ersteller.

### Gestaltung der Behandlungsvorgaben

Es wurde betrachtet, ob die Handlungsempfehlungen eine Trennung zwischen Handeln nach § 2a NotSanG und ÄLRD-Delegation erkennen ließen. So wurden diese als „getrennt“ verstanden, falls die einzelnen Algorithmen ausschließlich § 2a- oder ÄLRD-Delegationsmaßnahmen aufweisen. Enthält mindestens ein Algorithmus sowohl § 2a-Maßnahmen als auch delegierte Elemente, wurde dies als „kombiniert“ bezeichnet. Maßgeblich für die Bewertung war zunächst die Deklaration durch die Ersteller. Falls diese nicht vorlag, wurde die Einschätzung des Charakters der enthaltenden Elemente durch die Autoren herangezogen. Nichtdeklarierte bzw. als unklar charakterisierte Einzelmaßnahmen wurden bei der Bewertung außer Acht gelassen.

## Ergebnisse

### Eingeschlossene Behandlungsvorgaben

Landeseinheitliche Vorgaben ohne relevante regionale Abweichungen konnten für 11 Bundesländer identifiziert werden ([5–14]; Tab. 2). Für MV, SN und ST ist dabei eine gemeinsame Algorithmensammlung berücksichtigt [12]. Bezüglich der Länderkürzel siehe Tab. 3.

**Tab. 2:** Organisatorische und gesetzliche Rahmenbedingungen für Behandlungsvorgaben in den Bundesländern und für die Algorithmen des Deutschen Berufsverbands für den Rettungsdienst (DBRD). Die Tabelle stellt den Stand Juni 2022 dar. Bezüglich sich daraus ergebender Limitationen s. Abschnitt „Diskussion“

Bundesland	Regelungsebene	ÄLRD	Rechtsgrundlage ÄLRD-Delegation	BtM-Gabe durch NotSan
Baden-Württemberg	Landesweit [7]	Ja	Keine	Rechtf. Notstand
Bayern	Landesweit [6, 9]	Ja	Gesetzlich	Rechtf. Notstand + ÄLRD-Delegation
Berlin	Landesweit [5]	Ja	Gesetzlich	Nein
Brandenburg	Landesweit [11]	Ja	Gesetzlich	Nein
Bremen	Landesweit [8]	Ja	Keine	ÄLRD-Delegation
Hamburg	Landesweit [13]	Ja	Gesetzlich	Nein
Hessen	Regional [15–17]	Ja	Keine	Nein
Mecklenburg-Vorpommern	Landesweit [12]	Ja	Keine	Nein
Niedersachsen	Regional [18–20]	Ja	Keine	Rechtf. Notstand + ÄLRD-Delegation
Nordrhein-Westfahlen	Regional [21]	Ja	Keine	Unklar
Rheinland-Pfalz	Regional [22–27]	Ja	Gesetzlich	Nein
Saarland	Landesweit [14]	Ja	Gesetzlich	Nein
Sachsen	Landesweit [12]	Ja	Keine	Nein
Sachsen-Anhalt	Landesweit [12]	Ja	Gesetzlich	Nein
Schleswig-Holstein	Regional [28–30]	Ja	Gesetzlich	Rechtf. Notstand + ÄLRD-Delegation
Thüringen	Landesweit [10]	Ja	Gesetzlich	ÄLRD-Delegation
DBRD [3]	n. a.	n. a.	n. a.	Rechtf. Notstand

*n.* *a.* nicht anwendbar. *Rechtf. Notstand* Rechtfertigender Notstand. *ÄLRD* Ärztliche Leitung Rettungsdienst

**Tab. 3:** Abkürzungsverzeichnis der Bundesländer

Baden-Württemberg	BW
Bayern	BY
Berlin	BE
Brandenburg	BB
Bremen	HB
Hamburg	HH
Hessen	HE
Mecklenburg-Vorpommern	MV
Niedersachsen	NI
Nordrhein-Westfalen	NW
Rheinland-Pfalz	RP
Saarland	SL
Sachsen	SN
Sachsen-Anhalt	ST
Schleswig-Holstein	SH
Thüringen	TH

Für die restlichen 5 Länder flossen die Algorithmen aus je 3 RDB in die Auswertung ein (Tab. 2). In NW existiert für die Region Nordrhein ein RDB-übergreifendes Kompendium für den Rettungsdienst, welches jedoch den NotSan regional abweichende Befugnisse zuweist und daher als regional unterschiedlich klassifiziert wurde [21].

Zu 9 Ländern war zu jedem Krankheitsbild eine Behandlungsvorgabe zu finden. Unvollständige Behandlungsvorgaben im Sinne der Suchkriterien konnten für 3 Länder mit landeseinheitlichen Algorithmen (BY (*n* = 2 abgedeckte Krankheitsbilder), SL (*n* = 3), TH (*n* = 4)) und 4 Ländern mit regional abweichenden Vorgaben (HE: Frankfurt (*n* = 4), NI: Cuxhaven (*n* = 4), RP gesamt (*n* = 3) sowie Ludwigshafen und Südpfalz (*n* = 4), SH: Rettungsdienstkooperation in SH (RKISH) (*n* = 1)) eingeschlossen werden. Stellenweise existierten mehrere Algorithmen für ein Krankheitsbild, z. B. die Analgesie (DBRD, Nordrhein (Aachen, Kleve, Rhein-Kreis Neuss)) und den hypertensiven Notfall (DBRD, TH), welche alle mitbetrachtet wurden. Insgesamt wurden 112 Algorithmen und 403 Einzelmaßnahmen identifiziert und einzeln bewertet. Für HH konnten im Internet keinerlei Vorgaben gefunden werden. Die Algorithmen für HH wurden freundlicherweise auf Anfrage durch die ÄLRD für die Auswertung zur Verfügung gestellt.

### Voraussetzungen für die ÄLRD-Delegation

In allen Bundesländern sind ÄLRD eingerichtet. Demgegenüber konnte eine gesetzliche Verankerung der Delegation von heilkundlichen Maßnahmen durch die ÄLRD oder entsprechend verantwortliche Ärztinnen oder Ärzte nach § 4 Abs. 2 Nr. 2c NotSanG auf Landesebene zum Zeitpunkt der Datenerhebung in 9 Bundesländern identifiziert werden (Tab. 2).

Auch in den Ländern HE und NW, in denen kein gesetzlicher Auftrag an die ÄLRD oder andere Ärztinnen oder Ärzte zur Delegation von heilkundlichen Tätigkeiten an NotSan vorliegt, sind einzelne Behandlungsvorgaben von den Erstellern explizit als ÄLRD-Delegation deklariert (Tab. 4).

**Tab. 4:** Deklaration und objektivierbarer Charakter der einzelnen NotSan-Maßnahmen als § 2a-Maßnahmen (§ 2a) oder ÄLRD-Delegation (2c) nach Bundesländern und DBRD. Die Tabelle stellt den Stand Juni 2022 dar. Bezüglich sich daraus ergebender Limitationen s. Abschnitt „Diskussion“

Bundesland	Anzahl der Maßnahmen	Deklariert	Charakter	Konkordanz bewertbar	Konkordant (% bewertbar)
Baden-Württemberg	15	§ 2a: 9 (60 %) 2c: 0 (0 %) Nicht deklariert: 6 (40 %)	§ 2a: 6 (40 %) 2c: 2 (13 %) Unklar: 7 (47 %)	6 (40 %)	6/6 (100 %)
Bayern	8	§ 2a: 0 (0 %) 2c: 7 (88 %) Nicht deklariert: 1 (13 %)	§ 2a: 0 (0 %) 2c: 8 (100 %) Unklar: 0 (0 %)	7 (88 %)	7/7 (100 %)
Berlin	18	§ 2a: 4 (22 %) 2c: 6 (33 %) Nicht deklariert: 8 (44 %)	§ 2a: 0 (0 %) 2c: 9 (50 %) Unklar: 9 (50 %)	6 (33 %)	4/6 (67 %)
Brandenburg	12	§ 2a: 0 (0 %) 2c: 0 (0 %) Nicht deklariert: 12 (100 %)	§ 2a: 0 (0 %) 2c: 0 (0 %) Unklar: 12 (100 %)	0 (0 %)	n. a.
Bremen	25	§ 2a: 0 (0 %) 2c: 0 (0 %) Nicht deklariert: 25 (100 %)	§ 2a: 3 (12 %) 2c: 12 (48 %) Unklar: 10 (40 %)	0 (0 %)	n. a.
Hamburg	10	§ 2a: 0 (0 %) 2c: 10 (100 %) Nicht deklariert: 0 (0 %)	§ 2a: 0 (0 %) 2c: 9 (90 %) Unklar: 1 (10 %)	9 (90 %)	9/9 (100 %)
Hessen	53	§ 2a: 0 (0 %) 2c: 29 (55 %) Nicht deklariert: 24 (45 %)	§ 2a: 1 (2 %) 2c: 23 (43 %) Unklar: 29 (55 %)	17 (32 %)	17/17 (100 %)
Mecklenburg-Vorpommern, Sachsen, Sachsen-Anhalt	23	§ 2a: 23 (100 %) 2c: 0 (0 %) Nicht deklariert: 0 (0 %)	§ 2a: 3 (13 %) 2c: 0 (0 %) Unklar: 20 (87 %)	3 (13 %)	3/3 (100 %)
Niedersachsen	42	§ 2a: 0 (0 %) 2c: 0 (0 %) Nicht deklariert: 42 (100 %)	§ 2a: 8 (19 %) 2c: 15 (36 %) Unklar: 19 (45 %)	0 (0 %)	n. a.
Nordrhein-Westfahlen	66	§ 2a: 23 (35 %) 2c: 12 (18 %) Nicht deklariert: 31 (47 %)	§ 2a: 22 (33 %) 2c: 7 (11 %) Unklar: 37 (56 %)	29 (44 %)	27/29 (93 %)
Rheinland-Pfalz	26	§ 2a: 0 (0 %) 2c: 0 (0 %) Nicht deklariert: 26 (100 %)	§ 2a: 2 (8 %) 2c: 14 (54 %) Unklar: 10 (38 %)	0 (0 %)	n. a.
Saarland	16	§ 2a: 0 (0 %) 2c: 0 (0 %) Nicht deklariert: 16 (100 %)	§ 2a: 0 (0 %) 2c: 9 (56 %) Unklar: 7 (44 %)	0 (0 %)	n. a.
Schleswig-Holstein	36	§ 2a: 0 (0 %) 2c: 0 (0 %) Nicht deklariert: 36 (100 %)	§ 2a: 11 (31 %) 2c: 9 (25 %) Unklar: 16 (44 %)	0 (0 %)	n. a.
Thüringen	24	§ 2a: 12 (50 %) 2c: 7 (29 %) Nicht deklariert: 5 (21 %)	§ 2a: 0 (0 %) 2c: 13 (54 %) Unklar: 11 (46 %)	10 (42 %)	7/10 (70 %)
Zusammenfassung ohne DBRD	*374*	*§ 2a: 61 (16* *%)* *2c: 71 (19* *%)* *Nicht deklariert: 232 (62* *%)*	*§ 2a: 56 (15* *%)* *2c: 130 (35* *%)* *Unklar: 288 (77* *%)*	*88 (24* *%)*	*80/87 (92* *%)*
DBRD [3]	29	§ 2a: 18 (62 %) 2c: 0 (0 %) Nicht deklariert: 11 (38 %)	§ 2a: 11 (38 %) 2c: 0 (0 %) Unklar: 18 (62 %)	11 (38 %)	11/11 (100 %)
Zusammenfassung über alle	*403*	*§ 2a: 89 (22* *%)* *2c: 71 (18* *%)* *Nicht deklariert: 243 (60* *%)*	*§ 2a: 67 (17* *%)* *2c: 130 (32* *%)* *Unklar: 206 (51* *%)*	*99 (25* *%)*	*91/98 (93* *%)*

*n.* *a.* nicht anwendbar. *DBRD* Deutscher Berufsverband für den Rettungsdienst. Tabellenzellen der Zusammenfassugen sind zur besseren Abgrenzbarkeit kursiv dargestellt.

### Betäubungsmittel

Von den 16 Bundesländern sehen 6 die Verabreichung von im Betäubungsmittelgesetz regulierten Opioidanalgetika durch NotSan vor. In 3 Ländern existieren Vorgaben, die sowohl eine BtM-Anwendung in ÄLRD-Delegation als auch im rechtfertigenden Notstand vorsehen, einmal lediglich im rechtfertigenden Notstand und in 2 Bundesländern ausschließlich in ÄLRD-Delegation (Tab. 2).

### Deklaration und objektivierbarer Charakter als § 2a-Maßnahme oder ÄLRD-Delegation

Die Zuordnung durch die Ersteller zur Kategorie der § 2a-Maßnahmen oder einer ÄLRD-Delegation fand sich, wo vorhanden, in unterschiedlichen Konstellationen. Sie erfolgte für die gesamten Behandlungsvorgaben [3, 6, 7, 12, 13, 16] und/oder innerhalb der Algorithmen für einzelne Handlungsschritte durch textliche und/oder farbliche Kennzeichnung [5, 6, 10, 15, 21].

Von den 403 aus den verschiedenen Algorithmen extrahierten Einzelmaßnahmen waren 60 % durch die Ersteller nicht erkennbar als § 2a-Maßnahme und/oder ÄLRD-Delegation deklariert. Die restlichen Maßnahmen waren zu jeweils grob einem Fünftel den beiden genannten Verantwortungskategorien zugeteilt. Bei 6 Bundesländern konnte keinerlei Deklaration gefunden werden, wohingegen 4 Länder und der DBRD eine zumindest annähernd durchgehende Deklaration vorzuweisen haben (Tab. 4). Die gemeinsamen Behandlungsvorgaben für MV, SN und ST sind per se als § 2a-Maßnahmen vorgesehen, können jedoch explizit von den jeweils zuständigen ÄLRD auch delegiert werden [12]. In der Auswertung wurde jedoch nur die § 2a-Zuordnung berücksichtigt.

Für knapp die Hälfte der heilkundlichen Tätigkeiten konnte über eine objektive Bewertung der Charakter als § 2a- oder ÄLRD-delegierte Maßnahme festgestellt werden. Dabei überwog die Zuordnung als Maßnahme in ÄLRD-Delegation (33 %) gegenüber den § 2a-Maßnahmen (17 %). Zwischen den Bundesländern war eine große Heterogenität zu beobachten (Tab. 4).

Dort, wo ein Abgleich zwischen Deklaration und Charakter gezogen werden konnte, zeigte sich eine durchwegs hohe Konkordanz von im Mittel 93 %.

### Gestaltung der Behandlungsvorgaben

Vier Bundesländer, ein RDB und der DBRD präsentieren für die 5 beispielhaft ausgewerteten Krankheitsbilder reine § 2a-Algorithmen, 3 Länder und 2 RDB reine ÄLRD-Delegationsalgorithmen, und 2 Bundesländer und 2 RDB wiesen in individuellen Algorithmen eine Kombination von sowohl § 2a- als auch ÄLRD-Delegationsmaßnahmen auf. Ein getrenntes Nebeneinander von Behandlungsvorgaben nach § 2a NotSanG und ÄLRD-Delegation zeigte sich lediglich für Länder bzw. RDB ohne Deklaration der Maßnahmen durch die Ersteller, deren Eingruppierung auf der Bewertung des objektivierbaren § 2a- bzw. ÄLRD-Delegationscharakters der enthaltenen Elemente fußt (Tab. 5).

**Tab. 5:** Gestaltung der Behandlungsvorgaben für NotSan in Bezug auf die Kombination oder Trennung von § 2a- und ÄLRD-Delegationsmaßnahmen. Betrachtet wurden lediglich identifizierbare Algorithmen für die Krankheitsbilder akutes Koronarsyndrom, hypertensive(r) Notfall/Krise, Schlaganfall, Hypoglykämie und traumatisch bedingte Schmerzzustände. Die Tabelle stellt den Stand Juni 2022 dar. Bezüglich sich daraus ergebender Limitationen s. Abschnitt „Diskussion“

Bundesland	Gestaltung der Behandlungsvorgaben		
Baden-Württemberg	Reine § 2a-Algorithmen		
Bayern	Reine ÄLRD-Delegations-Algorithmen		
Berlin	Kombinierte § 2a-/ÄLRD-Delegations-Algorithmen		
Brandenburg*	n. a.		
Bremen*	Getrennt: Ein § 2a-Algorithmus 4 ÄLRD-Delegations-Algorithmen		
Hamburg	Reine ÄLRD-Delegations-Algorithmen		
Hessen	Frankfurt*: Getrennt: Ein § 2a-Algorithmus 2 ÄLRD-Delegations-Algorithmen Einmal uneindeutig	Main-Kinzig-Kreis: Reine ÄLRD-Delegations-Algorithmen	Landkreis Groß-Gerau: Reine ÄLRD-Delegations-Algorithmen
Mecklenburg-Vorpommern, Sachsen, Sachsen-Anhalt	Reine § 2a-Algorithmen (jedoch in einzelnen RDB auch als ÄLRD-Delegation möglich)		
Niedersachsen	NUN-Algorithmen*: Getrennt: 2 § 2a-Algorithmuen Ein ÄLRD-Delegations-Algorithmus 2 uneindeutig	Landkreis Cuxhaven*: Getrennt: Ein § 2a-Algorithmus 2 ÄLRD-Delegations-Algorithmen	Stadt Osnabrück*: Getrennt: Ein § 2a-Algorithmus 4 ÄLRD-Delegations-Algorithmen
Nordrhein-Westfahlen	Rhein-Kreis Neuss: Kombinierte § 2a-/ÄLRD-Delegations-Algorithmen	Kreis Kleve: Reine § 2a-Algorithmen	Städteregion Aachen: Kombinierte § 2a-/ÄLRD-Delegations-Algorithmen
Rheinland-Pfalz	Landesweite ÄLRD-Algorithmen*: Getrennt: Ein § 2a-Algorithmus 2 ÄLRD-Delegations-Algorithmen	Kaiserslautern und Trier*: Wie landesweite Algorithmen, zusätzlich 2 ÄLRD-Delegations-Algorithmen	Ludwigshafen und Südpfalz*: Wie landesweite Algorithmen, zusätzlich ein ÄLRD-Delegations-Algorithmus
Saarland*	Reine 2c-Algorithmen		
Schleswig-Holstein	Landesweite Ausbildungsalgorithmen*: Getrennt: 2 § 2a-Algorithmuen Ein ÄLRD-Delegations-Algorithmus 2 uneindeutig	Rettungsdienstkooperation in Schleswig-Holstein (RKiSH)*: Ein ÄLRD-Delegations-Algorithmus	Region Segeberg*: Getrennt: 2 § 2a-Algorithmen 2 ÄLRD-Delegations-Algorithmen Einmal uneindeutig
Thüringen	Kombinierte § 2a-/2c-Algorithmen		
DBRD [3]	Reine § 2a-Algorithmen		

*n.* *a.* nicht anwendbar, *ÄLRD* Ärztliche Leitung Rettungsdienst, *DBRD* Deutscher Berufverband für den Rettungsdienst

* Die Bewertung beruht auf dem objektivierten Charakter der enthaltenen Maßnahmen, da keine Deklaration durch die Ersteller vorlag

Für BB konnte keine Auswertung erfolgen, da neben der fehlenden Deklaration durch die Ersteller auch die Charakterisierung ausschließlich uneindeutige Ergebnisse erbrachte.

## Diskussion

Die vorliegende Auswertung zeigt in Bezug auf die Gestaltung der Behandlungsvorgaben für NotSan in der Bundesrepublik ein heterogenes Bild. Häufig variieren die Vorgaben von Landkreis zu Landkreis, eine ÄLRD-Delegation war bis zum Ende der Auswertungsperiode nicht erkennbar flächendeckend umgesetzt, und auch die Verabreichung von Betäubungsmitteln durch NotSan war offenbar zum Untersuchungszeitpunkt, der vor der Änderung des § 13 des Betäubungsmittelgesetzes und weiterer Gesetzesnovellierungen lag, nur in einzelnen Bundesländern etabliert.

Der aus Sicht der Autoren bemerkenswerteste Befund ist jedoch, dass für die Mehrzahl der für die Durchführung durch NotSan vorgesehenen Maßnahmen nicht erkennbar ist, wer die Verantwortung für die Indikationsstellung trägt. Konkret gesprochen: Handelt es sich um eine eigenverantwortlich durch NotSan zu indizierende Maßnahme nach § 2a NotSanG, oder sind die Behandlungsvorgaben von der ÄLRD auf deren Verantwortung an die NotSan delegiert?

Aufgrund der einleitend dargelegten Unterschiede zwischen eingeständiger Tätigkeit im Rahmen der Mitwirkung (ÄLRD-Delegation) und eigenverantwortlicher Heilkundeausübung nach § 2a NotSanG betonen sowohl der Wissenschaftliche Dienst des Bundestags [2] als auch das Bayerische Staatsministerium des Innern, für Sport und Integration [31] die Bedeutung einer Unterscheidung zwischen beiden Entitäten. Dennoch liegt bis dato keine empirische Auswertung darüber vor, inwieweit diese Differenzierung in der Praxis vollzogen wird.

### Eingeschlossene Behandlungsvorgaben

Für diese Auswertung wurden 5 Krankheitsbilder herangezogen, die häufig in Algorithmensammlungen zu finden sind. Laut Mann et al. ist dies für die hier untersuchten klinischen Bilder in 71–88 % der RDB der Fall. Davon abweichend finden sich für den Traumaschmerz lediglich in 52 % der ÄLRD-Zuständigkeitsbereiche entsprechende NotSan-Vorgaben [32]. Dennoch zählt die Trauma-Analgesie zu den am häufigsten durch NotSan angewendeten heilkundlichen Maßnahmen [33].

Die Tatsache, dass es zu einem Krankheitsbild keinen Algorithmus gibt, erlaubt allerdings nicht den Rückschluss, dass NotSan in diesen Fällen die Hände gebunden wären. Eine Tätigkeit nach § 2a NotSanG setzt ein Vorhandensein von Therapievorgaben nicht voraus. Beispielsweise sind für BY nur 2 Algorithmen aus der ÄLRD-Delegation in die Auswertung eingeflossen. Es bestehen jedoch weiterreichende, nichtalgorithmische Empfehlungen für die eigenverantwortliche Heilkundeausübung nach § 2a NotSanG, die nicht berücksichtigt werden konnten [34, 35]. Ähnliches gilt für das SL.

Aufgrund der großen Bedeutung für die Ausbildung schlossen wir auch die Musteralgorithmen des DBRD in die Auswertung ein. Die Ergebnisse des Pyramidenprozesses konnten nicht berücksichtigt werden, da diese nicht in Form von Behandlungsalgorithmen vorliegen [36].

### ÄLRD-Delegation

Für die Mehrheit der Bundesländer konnte eine gesetzliche Implementierung der ÄLRD-Delegation festgestellt werden, für 7 Länder jedoch nicht. In der zweiten Jahreshälfte 2022 hat zusätzlich der Landesausschuss für den Rettungsdienst in BW grünes Licht für die Delegation durch ärztliche Verantwortliche im Land gegeben [37, 38], ohne dass sich dies bis zum Ende des Erhebungszeitraums in Rettungsdienstgesetz oder Rettungsdienstplan niedergeschlagen hätte. Mittlerweile findet sich eine solche Regelung jedoch im § 20 des Rettungsdienstgesetzes Baden-Württemberg.

Auch wenn in den Rettungsdienstgesetzen von HE und NW keine explizite Beauftragung der ÄLRD zur Delegation nach § 4 Abs. 2 Nr. 2c NotSanG zu finden ist, steht diese Tatsache der ÄLRD-Delegation nicht grundsätzlich im Wege, so die Auffassung des Wissenschaftlichen Dienstes des Deutschen Bundestags [2].

Die untersuchten Krankheitsbilder umfassten für 6 Bundesländer eine explizite ÄLRD-Delegation, welche sich in 4 Ländern auch auf die BtM-Gabe erstreckte. Auch wenn dies für die anderen Länder nicht den Schluss zulässt, dass die ÄLRD-Delegation gar nicht zur Anwendung kommt, stellt sich die Frage nach den Ursachen für die fehlende Flächendeckung. Zum einen ist denkbar, dass eine ÄLRD-Delegation zwar nicht explizit als solche deklariert, aber dennoch intendiert ist. Andere Länder könnten der Auffassung folgen, dass durch die Heilkundeermächtigung nach § 2a NotSanG eine Delegation nicht länger erforderlich sei. Dadurch stünden den NotSan allerdings keine Handlungsoptionen für weniger kritische Notfälle, welche die Kriterien der Lebensbedrohung bzw. drohenden wesentlichen Folgeschäden nicht erfüllen, offen. Zu guter Letzt könnten die methodischen Hürden für ÄLRD-Delegationsalgorithmen abschrecken. Für die delegierende Ärztin bzw. den delegierenden Arzt stellt es durchaus eine Herausforderung dar, Delegationsvorgaben zu erstellen, die ohne wesentliches NotSan-Ermessen auf praktisch alle Fälle unter den Eingangskriterien anwendbar sind.

### Betäubungsmittel

Die eigenverantwortliche oder eigenständige Verabreichung von BtM durch NotSan ist in einer Minderheit der Länder in unterschiedlichen Konstellationen vorgesehen. Es steht zu vermuten, dass rechtliche und organisatorische Bedenken das eine oder andere Bundesland von einer entsprechenden Freigabe abhalten könnten.

Es sei angemerkt, dass zum Zeitpunkt der Datenerhebung eine BtM-Verabreichung durch NotSan noch nicht durch den im Jahr 2023 eingeführten § 13 Abs. 1b BtMG gerechtfertigt werden konnte, sondern auf den rechtfertigenden Notstand abstellen musste [31]. Da die Datenerhebung zum Zeitpunkt des Gesetzgebungsverfahrens bereits abgeschlossen war, konnte die BtMG-Novelle samt ihren Auswirkungen auf die jeweiligen Behandlungsvorgaben nicht mehr berücksichtigt werden. Die Sicherstellungsverantwortlichen für den Rettungsdienst werden zusammen mit den ÄLRD nun prüfen müssen, ob bereits bestehende Vorgaben zur BtM-Verabreichung den neuen Vorgaben entsprechen, und ob zusätzliche Freigaben entlang den neuen rechtlichen Möglichkeiten intendiert sind. Inwieweit die neue Rechtslage zu einer weiteren Verbreitung der BtM-Gabe durch NotSan führt, bleibt abzuwarten.

### Deklaration und objektivierbarer Charakter als § 2a-Maßnahme oder ÄLRD-Delegation

Lediglich 40 % der Maßnahmenvorgaben enthalten nach der vorliegenden Erhebung eine Aussage, ob sie nach § 2a NotSanG und/oder in ÄLRD-Delegation anzuwenden sind. Durch die Anwendung von objektivierbaren Kriterien sollte überprüft werden, inwieweit diese mit einer vorliegenden Deklaration der Ersteller im Einklang stehen, was zu einem weit überwiegenden Anteil der Fall war. Für Maßnahmen ohne Deklaration sollte durch die Punktebewertung eine Einordnung des Charakters als § 2a- oder ÄLRD-Delegationsmaßnahme ermöglicht werden.

Das hierfür entwickelte Punktesystem basiert u. a. auf den Ausführungen des Wissenschaftlichen Dienstes des Deutschen Bundestags zu den Merkmalen der eigenständigen und eigenverantwortlichen NotSan-Tätigkeit [2]. Es handelt sich dabei um den ersten beschriebenen Ansatz, die Zuordnung der heilkundlichen Tätigkeiten zu § 2a NotSanG oder ÄLRD-Delegation nach objektiven Kriterien nachzuvollziehen.

Die hohe Kongruenz bei den bewertbaren Maßnahmen zwischen der Deklaration durch die Ersteller und der Charakterisierung nach der vorliegenden Punktemethode wird dadurch methodisch begünstigt, dass die Bewertungsmatrix als ein Element die Deklaration der Ersteller selbst enthält.

### Gestaltung der Behandlungsvorgaben

Auch die Form der Behandlungsvorgaben variierte zwischen den Bundesländern. Es fanden sich für die 5 betrachteten Notfallsituationen reine § 2a-Maßnahmen- oder ÄLRD-Delegationskataloge, Algorithmen mit einer Kombination aus § 2a- und ÄLRD-Delegationselementen sowie ein Nebeneinander aus getrennten § 2a- und ÄLRD-Delegationsalgorithmen. Letzteres war jedoch ausschließlich für Bereiche feststellbar, in denen keine Zuordnung durch die Verantwortlichen erfolgte und statt dessen die oben genannte Methode zur Charakterisierung der Maßnahmen zur Anwendung gelangte. Daher ist eine Vergleichbarkeit für diese Bereiche nur bedingt gegeben.

### Limitationen

Die Auswertung stellt den Regelungsstand bis Juni 2022 dar. Im Rahmen der ständigen Weiterentwicklung des Systems Rettungsdienst haben sich seither relevante gesetzliche Änderungen ergeben, deren Auswirkungen nicht in die vorliegende Arbeit eingeflossen sind. Hierbei sind beispielhaft die Ergänzung von § 13 des Betäubungsmittelgesetzes sowie insbesondere die Novellierung des Rettungsdienstgesetzes Baden-Württemberg zu nennen, welche nunmehr die Grundlage für eine Delegation heilkundlicher Maßnahmen durch ärztliche Verantwortliche im Rettungsdienst gelegt hat. Diese Änderung bildet seither die Basis für eine Delegation der Standardarbeitsanweisungen und Behandlungspfade im Rettungsdienst einer 6 Bundesländer repräsentierenden Arbeitsgruppe [39]. Eine Wiederholung der Erhebung zum aktuellen Zeitpunkt würde daher in einzelnen Aspekten zu anderen Ergebnissen führen, ohne dass die Autoren die Grundaussage der Studie infrage gestellt sehen.

Diese Auswertung basiert mit Ausnahme von HH ausschließlich auf öffentlich zugänglichen Behandlungsvorgaben, sodass kein Anspruch auf Vollständigkeit der Datengrundlage erhoben wird. Für die RKISH sind die Vorgaben offensichtlich unvollständig öffentlich zugänglich. Daher konnte nur ein Algorithmus berücksichtigt werden. Von einer Anfrage bei den Verantwortlichen wurde abgesehen, da angenommen wurde, dass zumindest landesweit gültige Vorgaben öffentlich zugänglich seien. Eine solche Anfrage hätte letztlich auch einen Bias durch selektive Rückantwort nicht verlässlich ausgeschlossen.

Durch die Beschränkung auf 5 Krankheitsbilder und maximal 3 Behandlungsvorgaben pro Bundesland stellt diese Auswertung nur einen unvollständigen Ausschnitt der NotSan-Algorithmen dar, was die Repräsentativität unserer Ergebnisse einschränkt. Insbesondere die Auswahl von 3 RDB aus großen Bundesländern ist zwangsläufig lückenhaft und weniger als verallgemeinerbarer Querschnitt zu verstehen, sondern vielmehr als beispielhafter Beleg für regionale Unterschiede in den Behandlungsvorgaben in diesem Bundesland.

Eine Validierung der Punktematrix zur Objektivierung des § 2a‑Maßnahme/ÄLRD-Delegationscharakters der analysierten Maßnahmen wurde nicht durchgeführt und ist nach Ansicht der Autoren nicht möglich, da kein Goldstandard für eine derartige Bewertung existiert. Auch die isolierte Heranziehung der Einstellungen der bayerischen ÄLRD zur grundsätzlichen Delegierbarkeit schränkt die Aussagekraft der Methode ein, da sie aus einem bundeslandspezifischen Kontext heraus zu verstehen sind. Allerdings ist dies die einzige verfügbare systematische Quelle zu dieser Fragestellung, sodass deren Verwendung alternativlos erscheint. Einige der hier als nichtdelegierbar bewerteten Maßnahmen sind andererseits beispielsweise in Hamburg Bestandteil der ÄLRD-Delegation [13]. Die Ergebnisse aus dieser Punktebewertung sind somit lediglich als Anhaltspunkt zu verstehen.

### Sinnhaftigkeit einer Vereinheitlichung von Behandlungsvorgaben

Aus der in dieser Arbeit getroffenen Feststellung der hochgradigen Diversität von Behandlungsvorgaben über das Bundesgebiet drängt sich naturgemäß die Frage auf, ob nicht eine stärkere Vereinheitlichung sinnvoll wäre. Die Maximalvariante könnten bundeseinheitliche Vorgaben darstellen, wie sie auch bereits gefordert wurden [40, 41].

Solange eine Rettungsmittelbesatzung während ihrer Schicht unabhängig vom Einsatzort nach den ihr bekannten Behandlungsvorgaben agieren kann, stellen abweichende Vorgaben in benachbarten Bereichen zunächst keine grundsätzliche Einschränkung der Handlungsfähigkeit dar. Trotzdem können durch die folgenden Zusammenhänge Dilemmata entstehen.

#### Personalwechseldilemma

Bei einem Wechsel von NotSan in einen Bereich mit abweichenden Behandlungsvorgaben, etwa bei einem Arbeitsplatzwechsel oder einer entsprechenden Einteilung beim gleichen, RDB-übergreifend agierenden Arbeitgeber, ist eine Umstellung auf die dort geltenden abweichenden Vorgaben erforderlich. Die mutmaßlich größte Herausforderung dürfte sich dabei für Mitarbeitende von Leiharbeitsfirmen oder Personalbörsen ergeben.

#### Schnittstellendilemma

Auch bei der Zusammenarbeit mit anderen Einsatzbeteiligten (insbesondere dem NA) oder der Patientenübergabe an (oder Übernahme von) einer Klinik können sich Probleme durch unterschiedliche Erwartungshaltungen an die NotSan einstellen.

#### Alarmierungsdilemma

Eine weitere Herausforderung kann sich für die Leitstelle ergeben, wenn die Fähigkeit einer Rettungsmittelbesatzung, ein bestimmtes Meldebild abzuarbeiten, von den für das Rettungsmittel geltenden Behandlungsvorgaben abhängt. So wäre denkbar, dass der Rettungswagen (RTW) von Wache A einen Schmerzzustand gemäß ÄLRD-Delegation eigenständig (ohne NA) versorgen kann, während der RTW von Wache B hierfür einen NA benötigt. Gerade bei der Disposition von leitstellenfremden Rettungsmitteln entsteht somit eine unübersichtliche Ausgangslage.

#### Landesweite Vereinheitlichung als goldener Mittelweg?

Aus dem Gesagten ergibt sich, dass eine auf Ebene von Landkreisen variierende Regelungslandschaft ungünstig anmutet. Eine überregionale Vereinheitlichung erscheint daher sinnvoll. Andererseits profitieren NotSan am Bodensee wohl kaum spürbar davon, wenn deren Kollegin an der Kieler Förde nach den gleichen Vorgaben arbeitet. Unter der Berücksichtigung, dass Behandlungsvorgaben für NotSan idealerweise in das Gesamtkonstrukt Rettungsdienst eingebettet sind und in der Bundesrepublik für die Organisation des Rettungsdienstes die Bundesländer verantwortlich sind, stellt eine Vereinheitlichung der NotSan-Vorgaben auf Ebene der einzelnen Länder aus Sicht der Autoren einen guten Kompromiss dar.

## Fazit für die Praxis

- Zwischen den Bundesländern und auch teilweise innerhalb einzelner Länder besteht eine große Varianz bei den Behandlungsvorgaben für Notfallsanitäterinnen und Notfallsanitäter (NotSan).
- In der Mehrheit der für NotSan vorgesehenen Maßnahmen ist nicht ersichtlich, ob diese auf Grundlage von § 2a NotSanG oder einer Delegation durch die Ärztliche Leitung Rettungsdienst (ÄLRD) angewendet werden sollen, also wer die Indikationsverantwortung trägt. Eine entsprechende Deklaration durch die Ersteller könnte hier für mehr Klarheit bezüglich der Verantwortlichkeiten sorgen.
- Sowohl eine Delegation durch ÄLRD oder andere Ärztinnen oder Ärzte als auch eine Betäubungsmittelgabe ohne Arztanwesenheit ist nicht in allen Bundesländern etabliert.
- Eine Vereinheitlichung der NotSan-Behandlungsvorgaben zumindest bis auf Länderebene, die Implementierung der ÄLRD-Delegation und die Ermöglichung der Betäubungsmittelgabe für NotSan erscheinen sinnvoll.
- Die Auswirkungen hier nicht mehr erfasster und zukünftiger Änderungen der Rahmenbedingungen sollten in einer neuerlichen Untersuchung betrachtet werden.

## Data Availability

Die in dieser Studie erhobenen Datensätze können auf begründete Anfrage beim Korrespondenzautor angefordert werden.
